# Cod-Liver Oil

**Published:** 1849-04

**Authors:** 


*Cod-Liver Oil.*—The following account of the chief forms of disease in which it has been found useful, is taken from an essay on the "History of the Fish-Liver Oil," published in the *Gazette Medicale de Paris.*

*Chronic Rheumatism.*—According to Alexander, Knoodvon Helmendstreit, Amelung, Brefeld, Basse, Fehr, Galcoma, Mall, Moeunig, Munzenthalar, Michaelis, &c., who have all published their own observations concerning the fish-liver oil in chronic rheumatism, this medicine possesses such an efficacy in this disease that it surpasses in their eyes all the other remedies, without excepting the most lauded anti-rheumatics.

This opinion of different physicians, who have all experimented by themselves, cannot be taxed with exaggeration, if it is considered that amongst these cases there are found numerous instances of rheumatic patients being cured, who, after many years of suffering, and usage of all sorts of remedies, having lost their strength and despairing of cure, were completely cured by the aid of the fish-liver oil.

*Rheumatic Sciatica.*—The fish-liver oil did not prove less efficacious in this form of chronic rheumatism, which is generally distinguished by its obstinacy; this is verified by the observations of M. M. Knood von Helmendstreit, Rust, Amelung, Munzenthalar, Setttenger, and Spitter.

*Scrofulous Diathesis.*—Although there are various observations published in support of the excellence of this oil for certain severe forms of confirmed scrofula, it requires something, candidly speaking, which will prove its efficacy in the scrofulous diathesis with certainty. The cause of this doubt ought not to be looked for in this circumstance, that the liver oil is less applicable in the scrofulous diathesis than in certain of the more severe forms of scrofula, but that the greater part of physicians are in the habit of only publishing their observations of the most severe cases. But if we consider that the scrofulous diathesis is the principle from which emanates, by the accession of aggravating circumstances, all the numerous and often dangerous forms of scrofula, and that the liver oil is in our eyes a true specific for the more severe forms of this affection, it is evident that this medicine is that which ought to counteract this principle with most certainty. Such is the opinion of M. Brefeld and Dr. Galama, who say that the liver oil is the most efficacious remedy for the scrofulous diathesis, and for no matter what form of confirmed scrofula.

*Confirmed Scrofula.*—Amongst the facts relative to the use of the liver oil in some of the manifold forms in which confirmed scrofula is presented, the most remarkable are those which Drs. Brefeld and Roppe have made known, the result of which is that this medicine universally is fit for all forms and kinds of scrofula. The principal forms of scrofula in which it has succeeded are given below.

*Swelling of the Lymphatic Glands.*—Under this title we have only to do with the swelling of the superficial lymphatic glands, situated immediately under the skin, in the region of the throat, to the nape of the neck, armpits, or groins.

The fish-liver oil is considered a certain and infallible remedy for swellings of the lymphatic glands which appear, mostly, first under the form of hard unequal tumors, nearly immovable and insensible, but which afterwards, when inflammation has laid hold of the cellular tissue which surrounds them and the skin which covers them, become inflamed and suppurate in their turn. The cure always requires a much longer time where those swellings are connected with a confirmed scrofulous diathesis. This also can be advantageously influenced by the external use of the oil by frictions on the painful and inflamed tumours; this way of employing the medicine is that which has prevailed and which is recommended by the greater number of practitioners in this form of scrofula. But if the fish-liver oil is efficacious in swelling of the lymphatic glands of a scrofulous origin, it is absolutely useless in swellings of the same glands which are the consequence of small pox, measles, or scarlatina, or even those which are developed in the course of syphilis, or of a carcinomatous affection.

*Scrofulous Ulcers.*—The effect of this medicine is quicker and more remarkable in scrofulous ulcers, with fungous and irregular borders, generally so difficult to cure, which arise either from suppurative inflammation of lymphatic glandular swellings, or from the dissolution of those indurated strumous tumors which are found so often in subjects of a scrofulous constitution, in all parts of the body indifferently. It has the same effect also in different traumatic lesions which so frequently become the origin of ulcers in subjects of a full scrofulous habit. Dr. Brefield relies greatly on the external use of this oil, with which he prepares an ointment which he applies to the ulcers by means of a pledget. In one case, notwithstanding, treated by the oil internally, the result was as favorable. The strumous tumors which we have referred to above, and which ought to be distinguished from lymphatic glandular enlargements, are perfectly cured by the fish-liver oil, even after they have passed into the ulcerous state, provided that the oil be administered in proper time; it was the same in the case of the tumor being on the point of becoming an abscess. The tumors decreased during the internal and external administration of the medicine, and it seems they became dried up.

*Chronic Exanthemata.*—The fish-liver oil has been proved equally efficacious in the chronic exanthemata, which are developed under the influence of a scrofulous diathesis, whether they occupy parts of the body covered with hair or places which are destitute of it.

In this case, some say they have obtained the results from the internal use of this oil, while others pretend, on the contrary, to have obtained as good results by the external use of the same remedy. The usage of it externally, tried for the first time with success by Dr. Guerard, for scald head, is principally recommended by Dr. Brefeld, and who pretends, what is more, not to have obtained any good result from the internal use of the liver oil in the exanthematous form of scrofula.

The milky scurf, so often observed in ill-nursed children, in whom there have never before been observed any symptoms of scrofula, and which, according to Dr. Brefield, forms the transition of true scrofulous exanthemata; the exanthemata which are observed on the long-haired skin of young children, and which often envelope the whole face; scald head, which is not uncommon to see last till the age of puberty; and, finally, the scrofulous exanathemata which come out on every other part of the body, were quickly cured, according to Dr. Brefield, by the external use of the liver oil, and even after, in some cases, they had for a long time used the internal treatment in vain. Experience taught him that the use of the liver oil, either externally or internally, had no effect on malignant, hereditary, or contagious scald head, even when combined with oil of turpentine by the advice of Dr. Marteus; the same may be said of some psorical and symphilitic exanathemata.

Dr. Hauf reports a case of humid herpes causing an insupportable pruritus, which, after having resisted all sorts of remedies, was cured by the use of friction of fish-liver oil.

*Rachitis.*—The fish-liver oil is, without exception, the best remedy for rachitis, in all its stages, and under whatever form it presents itself; such is the nearly unanimous opinion of the German and Dutch Physicians, who affirm with one accord that it is much superior to any of the so-called antirachitic remedies. According to Dr. Schmidt, who has most insisted on the advantages of this medicine, in twenty-one rachitic patients which he had treated at the time when he made known his results, thirteen were cured, four were in process of being cured; as to the others, judging from the progress which they had made for the little time they were under treatment, a very favorable prognosis might be drawn.

In France, far from partaking of the enthusiasm of the German physicians for this medicine, they have kept on their guard, perhaps with an exaggerated distrust; its efficacy in rachitis has nevertheless appeared to some placed beyond doubt. We have said that M. Bretonneau and M. Trousseau, by their example, had obtained good results. It is in these terms that Professor Trousseau expresses himself on this subject: "We have often obtained cures, the rapidity of which surpassed our expectation.

Sometimes, after four days of treatment, the sharp pains which the children felt in all their limbs, ceased; and the bones which could be bent, acquired, at the end of five days, a considerable solidity.'

*General conclusions.*—Chemical researches have taught us that the fish-liver oil ought to be considered as a very compound medicine. Greasy, neutral matter, bilious matter, iodine, phosphorus, each of them, well known as possessing great therapeutic efficacy—also a certain number of organic elements, such as butyric acid, gaduine, and some others, the medical action of which is less known—finally, various inorganic salts, as the phosphate and sulphate of lime, chloride of lime, phosphate and sulphate of magnesia, are the substances of which it is composed.

But it may be asked, to which of these components does the oil owe its special virtues? Is it to the iodine, fatty matters, phosphorus, or other principles?

If the diseases, for which the liver oil is administered with success, be duly reflected upon, it cannot escape any one, that there are in each of them various indications to fulfil to obtain a cure. For the most part, there is debilitated digestion to be excited, nutrition to be regulated, secretions to be re-established, and the lymphatic system to be stimulated; while, on the other hand, the modifying of the organic nervous system, is presented as one of the most important indications to be fulfilled. Neither the bilious matter, nor the fatty matter, nor the iodine, nor any other principle, whatever it may be, taken alone, is capable of satisfying at the same time, all these indications, and it is not to any of these substances in particular, that the fish-liver oil owes its medicinal properties, and the faculty of fulfilling so different and so numerous indications. But it is by the union and co-operation of, if not all, at least the greater number of these substances.

In this state of things, the active principle of the fish-liver oil cannot be discussed in particular, like the active principle of cinchona; but attention ought to be paid, if not to all, at least to the principal elements of the oil, as each of them satisfying special indications which the diseases for which this medicine has been proved efficacious, present.

The medical researches having proved that the black fish-liver-oil is more efficacious in rheumatism and scrofula than the other species, and the chemical researches having shown, on the other hand, differences, if not qualitative, at least quantitative, between the three kinds of oil examined, it follows that the principles that are in greater proportions in the black oil than in the other two kinds, ought to be considered as those which best fulfil the principal indications. Therefore it is not the neutral fatty matters, which are found in nearly equal quantities in the three species, nor the iodine, nor the phosphorus, nor the organic salts, which are found in greater quantity in the pale oils than in the black oil, which can be considered as more efficacious than the other principles for the cure of rheumatism and scrofula. It appears, then, that it is to the bilious matter and butyric acid, rather than the other principles, that the greater part of the therapeutic effect can be principally attributed, for they are the substances which are found in the greatest quantity in the variety of oil proved to be the most active.

As to the matter unknown up to this time, and which M. Jough first proved the existence of, in the product of the analysis of the different species of Gadus, and to which he applied the name of Gadinæ, it does not appear, on account of its insolubility, at least in the condition in which it was examined, to have a right to be considered as an active principle of the fish-liver oil.—(*Gazette Medicale, and Dublin Med. Press.*

Dr. Bennett considers that the therapeutic action of cod liver oil is due to its fatty composition, and its being perhaps more easily assimilated than other fats. He believes that in rheumatic and tubercular affections, the albuminous compounds are in excess, and the oily compounds deficient; that, therefore, the most rational treatment is to supply the deficient oily matters directly. He explains the failure of other oils to effect benefit, which might be expected, if the fatty matter is the active principle, upon the supposition that other oils, such as olive oil, are purgative. The author proceeds to state that he thinks cod liver oil is destined, in the hands of the rational practitioner, "to be an important means of curing a class of diseases hitherto considered of the most dangerous and fatal character."

Speaking of the effect of this oil in phthisis, Dr. Bennett's testimony is greatly in its favor; and, in fact, it may now be satisfactorily demonstrated that there is no medicine or system of treatment which holds out so much encouragement in the management of consumptive cases.—("*On Cod Liver Oil,*" *Edinburgh,* 1848; *and Monthly Journal, May,* 1848.)—*Ranking's Report on Pract. Med. in Abstract, vol. vii.*

